# A case of acanthosis nigricans–like mycosis fungoides

**DOI:** 10.1016/j.jdcr.2021.08.032

**Published:** 2021-09-08

**Authors:** Dong Joo Kim, Sam Van Truong, Aimee Paik, Christina Kraus, Ronald M. Harris, Ronald J. Barr, Kenneth G. Linden, Janellen Smith

**Affiliations:** a Department of Dermatology, University of California, Irvine, Irvine, California; b Laguna Pathology Medical Group, Laguna Beach, California; c The Chao Family Comprehensive Cancer Center, University of California, Irvine, Irvine, California

**Keywords:** acanthosis nigricans, acanthosis nigricans–like, cutaneous T-cell lymphoma, mycosis fungoides, papillomatous, paraneoplastic, vegetating, AN, acanthosis nigricans, MF, mycosis fungoides

## Introduction

Acanthosis nigricans (AN) is a common skin condition characterized by symmetric, hyperpigmented, velvety skin thickening most commonly involving the neck, axillae, and other intertriginous areas. AN can be acquired or inherited and is most often associated with obesity, insulin resistance, and other endocrinologic and metabolic disorders. Infrequently, AN may represent a paraneoplastic (or malignant) phenomenon, usually associated with underlying gastrointestinal malignancies, especially gastric adenocarcinomas. However, rare cases of AN secondary to underlying cutaneous T-cell lymphoma have been described.^1^, ^2^, ^3^, ^4^ Complicating matters, an exceedingly rare, vegetating/papillomatous variant of mycosis fungoides (MF), referred to as “AN-like MF” has been reported.^2^^,^^5^, ^6^, ^7^ MF is the most common form of cutaneous T-cell lymphoma and the most prevalent form of primary cutaneous lymphoma overall. The clinical manifestations of MF have been well-documented and are heterogeneous, often mimicking other skin conditions.^8^ In addition to classical MF (Alibert-Bazin type), numerous other variants have been described.^9^^,^^10^ Herein, we present a case of AN-like MF with possible associated paraneoplastic AN.

## Case report

A 35-year-old Asian woman presented with a pruritic rash of 2 years' duration that began a few weeks after delivery of her second child. The lesions started as skin-colored papules and plaques scattered on the trunk that coalesced and progressed to involve the neck and extremities. Over time, the lesions became increasingly raised, discolored, and coarse. The patient had gestational diabetes during her second pregnancy, which was well controlled with metformin and resolved promptly postpartum. She was otherwise healthy, with no history of persistent endocrinologic diseases, malignancies, chronic skin diseases, or other health conditions requiring long-term medications.

**Fig 1:**
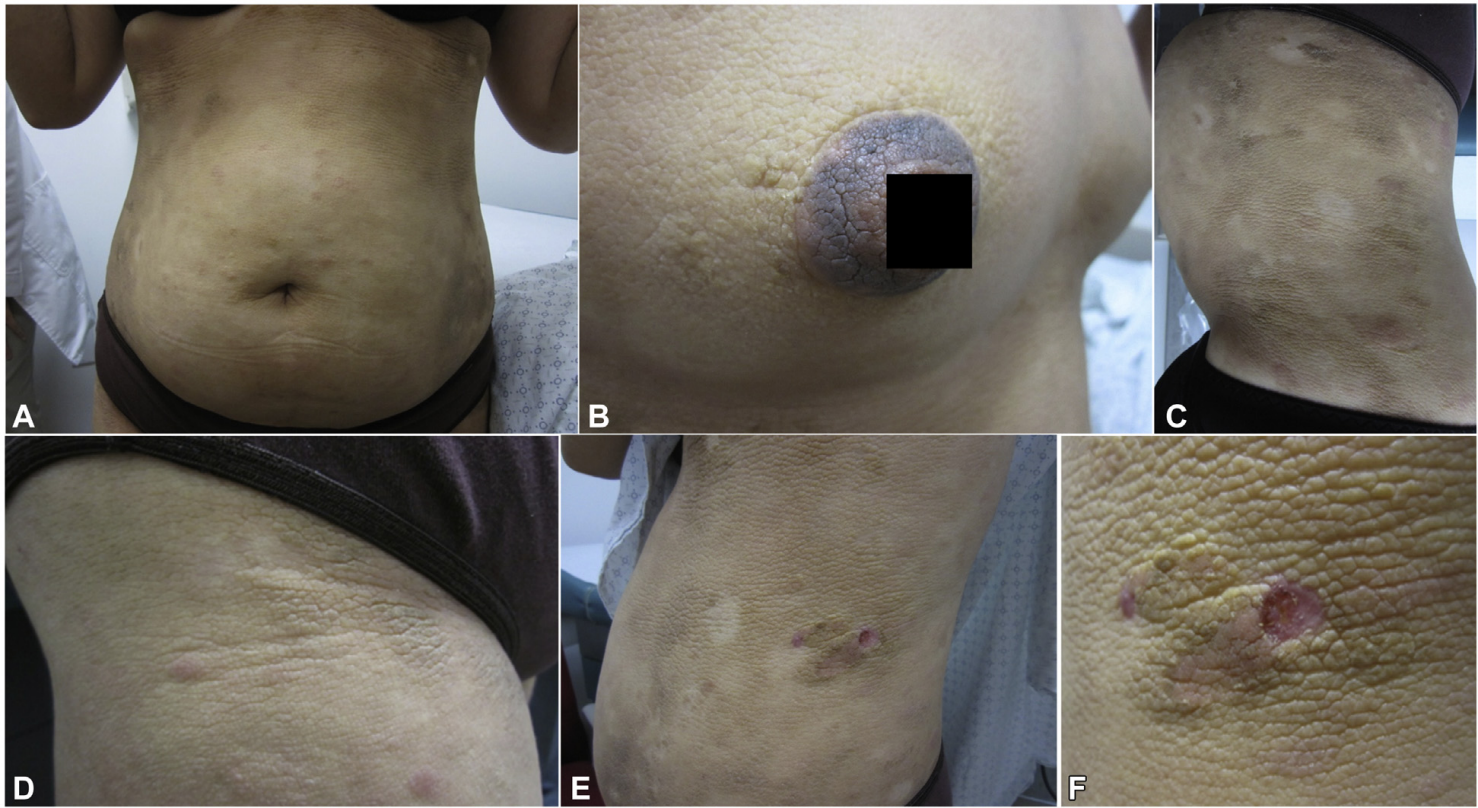
Acanthosis nigricans–like mycosis fungoides, clinical presentation. Hyperpigmented, thickened, velvety plaques with exaggerated skin markings on the (**A**) chest, (**B**) areolae, (**C**, **D**) abdomen, and (**E**, **F**) flanks. Areas of sparing were admixed with scattered erythematous, hypopigmented and hyperpigmented patches.

On examination, the patient had diffuse areas of thickened, velvety, hyperpigmented, and violaceous skin with exaggerated skin markings on the trunk, inguinal folds, and proximal aspect of thighs (Fig 1). Areas of sparing were admixed with scattered erythematous, hypopigmented and hyperpigmented patches that highlighted the color variegation of her disease process. No hepatosplenomegaly or lymphadenopathy was observed, and complete blood cell count, comprehensive metabolic panel, hemoglobin A1C, and thyroid function tests were normal. Computed tomography imaging of the neck, chest, abdomen, and pelvis was negative for lymphadenopathy and was otherwise unremarkable.

**Fig 2:**
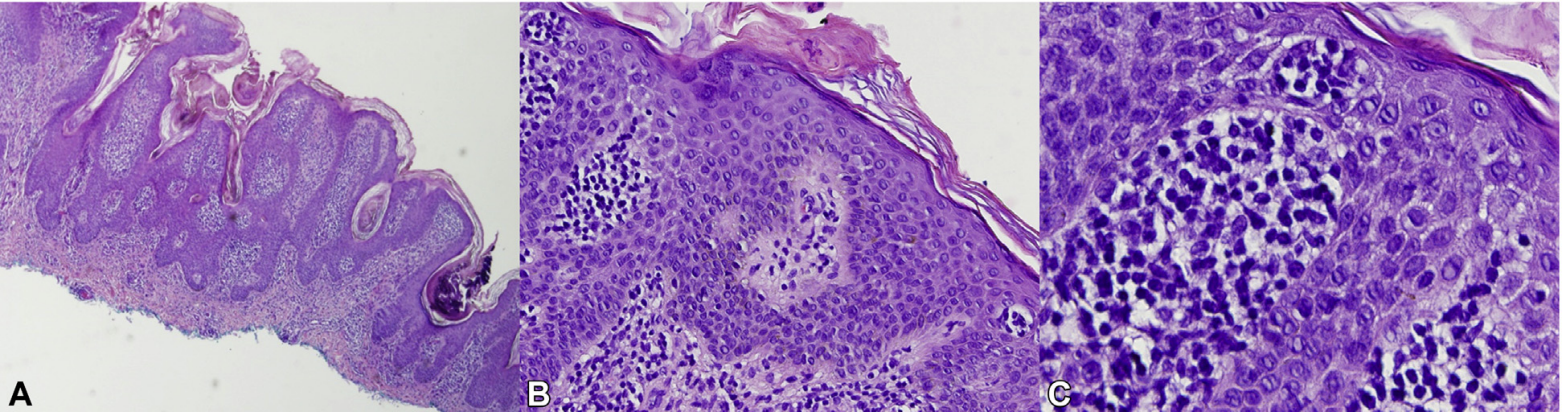
Acanthosis nigricans–like mycosis fungoides, histopathologic manifestations. Multiple skin biopsies from different sites were obtained. **A**, Shave biopsy from the left side of the chest showed hyperkeratosis and papillomatosis with lacelike interdigitation and pseudocystic invagination in the stratum malpighii. **B** and **C**, There was epidermotropism of atypical lymphocytes as well as Pautrier microabscesses in the presence of mild-to-moderate spongiosis. Atypical lymphocytes were lined up along the basal layer, and there was wiry fibrosis of the dermal papillae, which housed a dense superficial perivascular and lichenoid infiltrate of atypical lymphocytes.

**Fig 3:**
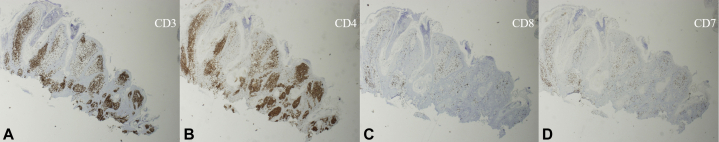
Acanthosis nigricans–like mycosis fungoides, immunohistochemistry. Immunohistochemical studies demonstrated a (**A**) CD3^+^, (**B**) CD4^+^, (**C**) CD8^−^, and (**D**) CD7^−^ immunophenotype with a markedly elevated CD4:CD8 ratio. (**A**, CD3 stain; **B**, CD4 stain; **C**, CD8 stain; **D**, CD7 stain.)

**Fig 4:**
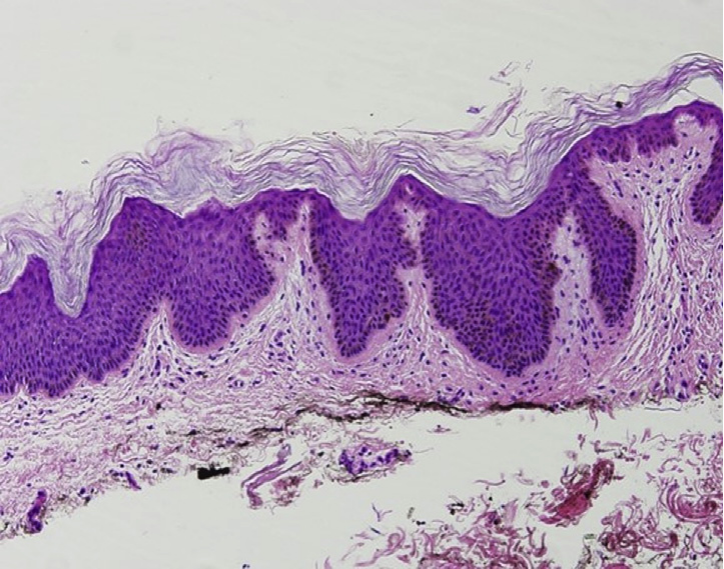
Acanthosis nigricans. Shave biopsy from the left axilla showed hyperkeratosis, papillomatosis, and intervening “valleys” with underlying acanthosis and basilar hyperpigmentation consistent with acanthosis nigricans while lacking the features of mycosis fungoides.

Multiple skin biopsies from different sites were performed. Shave biopsies from the left side of the chest (Fig 2, *A*) and the left flank (not shown) revealed hyperkeratosis, papillomatosis with lacelike interdigitation, and pseudocystic invaginations into the stratum malpighii. The epidermis was notable for benign cellular hyperplasia with mild-to-moderate spongiosis and epidermotropism of slightly enlarged, hyperchromatic lymphocytes with cerebriform nuclei forming variably-sized collections (Pautrier microabscesses) (Fig 2, *B* and *C*). These atypical lymphocytes were also present along the basal layer (some surrounded by clear halos) and as dense lichenoid and superficial perivascular infiltrates housed by wiry fibrosis of the papillary dermis. Immunohistochemical studies demonstrated a CD3^+^/CD4^+^/CD8^−^/CD7^−^ immunophenotype with markedly elevated CD4:CD8 ratio (Fig 3, *A* to *D*). Additionally, immunohistochemical staining was Bcl-2^+^, CD2^+^, CD5^+^, CD43^+^, CD20^−^, and CD30^−^. T-cell receptor gene rearrangement studies showed a clonal T-cell receptor β/γ gene rearrangement. Flow cytometry revealed no immunophenotypic abnormalities. A shave biopsy from the left axilla (Fig 4) showed hyperkeratosis, papillomatosis, and intervening “valleys” with underlying acanthosis and basilar hyperpigmentation, consistent with isolated AN.

The patient received a diagnosis of AN-like MF with concomitant non-MF AN and was started on topical clobetasol and twice weekly narrowband ultraviolet B phototherapy. Over a period of 6 years, the patient was transitioned from light therapy (partial response) to oral methotrexate titrated up to 22.5 mg weekly (partial response) to oral bexarotene titrated up to 375 mg/m^2^/d, eventually tapering off of methotrexate given clearance of AN and AN-like MF plaques on bexarotene (complete response). Although her AN-like MF lesions resolved, new, scattered, flesh-colored to pink nodules subsequently developed on her nose, axillae, and inner thighs, biopsies of which were consistent with tumor-stage MF (CD2^+^/CD3^+^/CD4^+^/CD5^+^/CD7^−^ admixed with reactive CD8^+^ lymphocytes and CD30 highlighting only scattered large cells less than 25% of the population). She was restaged as stage IIB by Hematology/Oncology, and oral bexarotene was titrated up to 375 and 450 mg/m^2^ on alternate days, in addition to topical corticosteroids and adjunctive intralesional triamcinolone injections (30-35 mg/mL), with partial response.

## Discussion

The vegetating/papillomatous variant of MF (“AN-like MF”) is a rare subset of the disease (Table I).^2^^,^^5^, ^6^, ^7^ In a limited number of cases, there does not appear to be any predilection for age, race, or sex. Flexural, umbilical, and areolar skin involvement predominates, representing a departure from the traditional buttocks and covered distribution of classical MF. Clinically, this subset of MF presents as velvety, hyperpigmented, polygonal plaques with variable patches of erythema interspersed between the lesions. Over time, some lesions may become more pronounced and even nodular. Pruritus may be a prominent feature. Histologic examination of lesional skin reveals features of both AN and MF. The treatment of AN-like MF is anecdotal, and topical corticosteroids, topical nitrogen mustard, psoralen plus ultraviolet A photochemotherapy, and electron beam therapy have been used successfully. Our patient improved with topical corticosteroids, narrowband ultraviolet B, oral methotrexate, oral bexarotene, and intralesional triamcinolone injections, further expanding the therapeutic ladder for AN-like MF.

**Table I:** Summary of potential acanthosis nigricans–like mycosis fungoides in the literature

Characteristic	Kanitakis et al^6^ (1977)	Willemze et al^5^ (1985)	Neill et al^2^ (1985)∗	Puig et al^7^ (1996)	Cheng et al^4^ (2015)∗	Our patient (2015-2020)
Ethnicity	White	Black	White	White	Not available	Asian
Age, y	20	62	55	58	67	36
Sex	Female	Male	Male	Female	Male	Female
Personal history of diabetes	Not reported	Not reported	None	None	Type II diabetes mellitus	Gestational diabetes
Family history of diabetes	Not reported	Not reported	Not reported	Not reported	Not reported	Type II diabetes mellitus (father)
Concomitant paraneoplastic AN	No	No	Yes	Yes	Yes	Favored
Concomitant classical MF	No	Yes	Yes	Yes	Not reported	Tumor-stage MF subsequently developed
Distribution	Neck, trunk, nipples/areolae, axillae, and inguinal folds	Neck, axillae, inguinal folds, antecubital folds, and right leg	Axillae, nipples, umbilicus, and groin	Antecubital folds, axillae, groins, popliteal fossae, intergluteal, and periumbilical	Axillae, neck, and lower portion of legs	Axillae, chest, areolae, abdomen, flanks, inguinal folds, proximal aspect of thighs, and forearms
Treatment (response)	Topical betamethasone 8 mg/d for 20 d (“much improved”)	Electron beam therapy 4 MeV, 30 Gy, whole body (complete); unspecified topical corticosteroid for recurrence (complete)	PUVA alone, unspecified (none); topical nitrogen mustard unspecified plus PUVA unspecified (partial)	Topical triamcinolone 0.1% and PUVA (complete)	Planned for PUVA but lost to follow-up	Topical clobetasol (partial), twice weekly NBUVB (partial), oral methotrexate 22.5 mg weekly (partial), oral bexarotene 375 mg/m^2^/d (complete)

*AN*, Acanthosis nigricans; *MF*, mycosis fungoides; *NBUVB*, narrowband ultraviolet B phototherapy; *PUVA*, psoralen plus ultraviolet A photochemotherapy.

∗ AN-like MF was not specifically mentioned despite the presence of biopsy-proven features of both AN and MF overlapping within the same lesion.

Interestingly, AN has been described as a paraneoplastic phenomenon in cases of MF, in both the classical^1^^,^^3^^,^^4^ and vegetating/papillomatous types.^2^^,^^7^ The concomitant presentation of AN (benign or paraneoplastic) and AN-like MF makes diagnosis and distinction between the 2 entities difficult. A complete evaluation for the other more common etiologies of AN must be performed. In cases of AN with negative preliminary workup and unknown etiology, a skin biopsy of an existing dermatosis may prove useful, as MF is a great imitator and early lesions are frequently misdiagnosed.^8^ Furthermore, biopsy of a lesion itself may be considered, especially when the distribution is widespread or atypical, as this may in fact represent AN-like MF. This distinction is important, because the management of MF and AN is different. In our patient, the concomitant presentation of biopsy-proven AN in the setting of AN-like MF could represent idiopathic AN that arose incidentally with her AN-like MF, AN associated with her prior gestational diabetes and/or pregnancy, early stages of AN-like MF that had not yet fully evolved histologically, or malignant AN secondary to her underlying AN-like MF. We favored the latter, paraneoplastic phenomenon, given her negative systemic workup and resolution of AN lesions after treatment of her AN-like MF, although this hypothesis cannot be fully proven without serial, longitudinal biopsies, which proved burdensome for the patient. Our report is limited by the absence of T-cell receptor gene rearrangement studies on the left axillae biopsy specimen of isolated AN (Fig 4), which might have revealed an underlying AN-like MF that was not overtly evident on routine histopathology.

## Conflicts of interest

None disclosed.
